# A 6-Year-Old Boy with COVID-19-Positive Pleural Effusion and Kawasaki-Like Features

**DOI:** 10.1155/2021/8832826

**Published:** 2021-03-17

**Authors:** Sedigheh Yousefzadegan, Ramin Zare Mahmoudabadi, Golnaz Gharehbaghi

**Affiliations:** ^1^Department of Pediatric Pulmonology, Firoozabadi Clinical Research Development Unit (FACRDU), Iran University of Medical Sciences, Tehran, Iran; ^2^Department of Pediatrics, Pediatric Intensive Care Unit, Firoozabadi Clinical Research Development Unit (FACRDU), Iran University of Medical Sciences, Tehran, Iran; ^3^Department of Pediatrics, Ali Asghar Children's Hospital, Iran University of Medical Sciences, Tehran, Iran

## Abstract

The ongoing outbreak of the novel coronavirus (SARS-CoV-2) has exposed many pediatric patients from around the world to coronavirus disease. Although pleural effusions are classified as atypical features of COVID-19 infection, we report a 6-year-old boy who had a positive IgG antibody ELISA test for COVID-19 and presented with respiratory distress, bilateral pleural effusions, and signs and symptoms of multisystem inflammatory syndrome. The RT-PCR test of the pleural fluid specimen was positive for novel coronavirus. To our knowledge, this is the first pediatric report of a COVID-19-positive pleural fluid.

## 1. Introduction

The ongoing outbreak of the novel coronavirus (SARS-CoV-2) has exposed nearly 90 million people to coronavirus disease (COVID-19), including many pediatric patients all around the world [1]. Because of the milder nature and lower incidence of the disease in children, there is uncertainty about the symptomatic diagnosis and clinical management of COVID-19 pneumonia in pediatric patients [2]. Multisystem Inflammatory Syndrome in Children (MIS-C) is a condition where many organs are involved with COVID-19 infection, and it causes some Kawasaki symptoms but did not fulfill the criteria of Kawasaki. Typical ground-glass opacities in lung parenchyma could be a part of MIS-C, but other forms of pulmonary involvement such as pleural effusion is unlikely. Herein, we introduce one case of MIS-C syndrome with pleural effusion. To our knowledge, this is the first pediatric report of COVID-19-positive pleural fluid.

## 2. Case Presentation

A 6-year-old boy presented with persistent fever for five days, diarrhea, dry cough, and gradual onset of respiratory distress. Two weeks before the start of his symptoms, he was in contact with his aunt just before she tested positive for COVID-19. He was a healthy child with no history of previous medical conditions or hospital admission.

**Figure 1:**
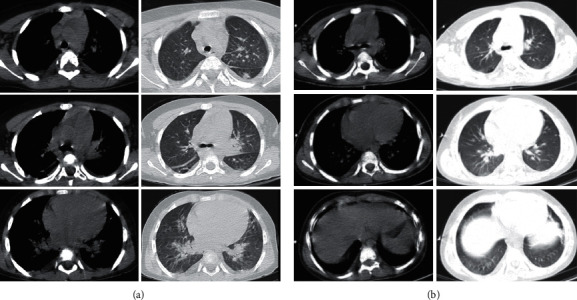
(a) The computed tomography (CT) scan depicts increasing vascular marking, interlobular septal thickening, ground-glass opacities, and mild bilateral pleural effusion. (b) A second computed tomography of the patient is shown here. There is no evidence of pleural effusion or pleural thickening, and the ground-glass opacities got almost clear.

On presentation, he was admitted to the pediatric intensive care unit (PICU) because of tachypnea (respiration rate = 75 beats per minute), subcostal retraction, and hypoxemia (oxygen saturation = 80% in room air). His chest computed tomography (CT) scan revealed bilateral focal subpleural opacities and bilateral mild pleural effusion (Figure 1(a)). He received 5–8 liters/minute of 100% oxygen with a mask because of respiratory distress, and his oxygen saturation rate was 88–91% at presentation. His condition improved with oxygen therapy without requiring intubation.

In addition to starting broad-spectrum antibiotics (ceftriaxone and vancomycin), due to suspicion of bacterial coinfection, the patient began treatment for COVID-19, according to the treatment protocol for COVID-19 issued by the Iranian National Health Commission (version 6), with hydroxychloroquine (5 mg/kg/day) and azithromycin (10 mg/kg/day) together with heart monitoring due to clinical features and contact history. His first nasopharyngeal reverse transcription-polymerase chain reaction (RT-PCR) test for COVID-19 that was taken on admission was negative.

On the 3rd day after admission, the patient demonstrated Kawasaki-like features: fever plus bilateral nonpurulent conjunctivitis, cervical lymphadenopathy, anemia, hypoalbuminemia, and elevated alanine aminotransferase (ALT). However, no other skin rash or mucosal changes were evident. The patient's echocardiography was normal. Because of the normal echocardiography, mild Kawasaki symptoms, and controversial treatment protocols for Kawasaki-like features in COVID-19 disease, intravenous immunoglobin or glucocorticoids were not started. There was also an elevated serum d-dimer level in favor of hypercoagulability state, so injection of enoxaparin was started. On the 5th day after admission, an enzyme-linked immunosorbent assay (ELISA) test was carried out on the patient's blood and revealed that his SARS-CoV-2 IgG was 10-fold higher than the sample cutoff but IgM was undetectable. His laboratory data are listed in Table 1.

Given the continuation of fever and respiratory distress on the 6th day after admission, antibiotics were stepped up to meropenem and vancomycin, and lung ultrasonography was carried out. Pleural fluid was aspirated and found to be positive for COVID-19 based on RT-PCR. The pleural fluid smear and culture came back negative. A second RT-PCR test was performed on a nasopharyngeal specimen that came back positive for COVID-19. On the 7th day after admission, a second chest CT scan, with low-risk protocol, was performed to rule out possible complications. The CT scan showed relatively resolving pulmonary lesions and pleural effusion (Figure 1(b)).

Beginning on the 6th day after admission (11th day of fever onset), the patient's fever became milder and his conjunctivitis resolved. After 14 days, the fever resolved completely. On the 11th day after admission, a second ELISA test on his blood was performed that was strongly positive for IgG and undetectable for IgM. The patient was discharged from the hospital after 18 days of hospitalization in good condition.

## 3. Discussion

This is an interesting informative case of MIS-C who was admitted with acute signs of pulmonary and autoinflammatory involvement. Kawasaki-like features were accompanied by an unusual sign of pleural effusion in the chest CT scan that was positive for the novel coronavirus. It is possible that this presentation could be explained by cytokine storm and subsequent multisystem inflammatory syndrome as it completes the WHO criterion on this matter [3]. Considerable IgG titers in two tests obtained on separate days with an 8-day interval and an undetectable IgM on both tests were not compatible with the fact that the patient was in the acute phase of the disease when he was admitted and it may be related to the seroconversion period in pediatric patients infected with COVID-19. There are several clinical and laboratory characteristics of COVID-19 patients that indicate cytokine storm syndrome in children: cytopenia and elevated ESR, CRP, and IL-6 [4–6]. In addition, the cytokine storm in COVID-19 patients may occur rapidly, with immune cells attacking the lungs soon after multisystemic inflammatory syndrome [3, 7]. Both of these mentioned observations are consistent with our case.

Our case presented with bilateral mild exudative pleural effusion that was positive for COVID-19 by RT-PCR. According to the International Expert Consensus Statement on Chest Imaging in Pediatric COVID-19 Patients, pleural effusions are classified as atypical features of COVID-19 infection [4]. It may be predictive of a worse prognosis and can indicate bacterial superinfection in COVID-19, so we started wide-spectrum antibiotics for him, but infection markers were not suggestive of bacterial involvement. There are only few cases of COVID-19-positive pleural fluid in adults, for example, a severely ill 80-year-old patient [5]. To our knowledge, this is the first report of COVID-19-positive pleural fluid in a pediatric patient.

## Figures and Tables

**Table 1:** Laboratory results in the 1st, 3rd, 6th, and 14th days after admission.

Measure	1st day	3rd day	6th day	14th day
White cell count (×10^9^/L)	8	9.2	8.59	11.4
Lymphocyte (%)	8.5	4	25	23
Hemoglobin (g/dL)	10.5	9.6	8.8	10.3
Platelet count (×10^9^/L)	181	326	464	620
C-reactive protein (mg/dL)	127	84	53	6
Erythrocyte sedimentation rate (mm/h)	—	65	83	—
Albumin (g/dl)	3.2	2.6	2.3	4
Lactate dehydrogenase (LDH) (U/L)	477	—	507	
Alanine aminotransferase (U/L)	18	88	80	35
INR	—	1	—	
Partial thromboplastin time (s)	—	40	—	
D-Dimer	—	1600	620	—
Blood culture	Negative	—	Negative	
Pleural fluid analysis^*∗*^	Glucose: 106 (mg/dL), protein: 2.4 (mg/dL), LDH: 254 (U/L), WBC: 3000 (cells/*μ*L), lymphocytes: 20 (%), neutrophils: 80 (%), and RBC: 12500 (cells/*μ*L)			

^*∗*^Values obtained on the 6th day after admission. A blood sample was taken at the same time: albumin (g/dL): 2.9, total protein (g/dL): 4.5, lactate dehydrogenase (U/L): 415.

## Data Availability

All data are available from the corresponding author on request.
